# Comparative genomics of plant pathogenic *Diaporthe* species and transcriptomics of *Diaporthe caulivora* during host infection reveal insights into pathogenic strategies of the genus

**DOI:** 10.1186/s12864-022-08413-y

**Published:** 2022-03-03

**Authors:** Eilyn Mena, Silvia Garaycochea, Silvina Stewart, Marcos Montesano, Inés Ponce De León

**Affiliations:** 1 Departamento de Biología Molecular, Instituto de Investigaciones Biológicas Clemente Estable, Avenida Italia 3318, CP 11600 Montevideo, Uruguay; 2 Instituto Nacional de Investigación Agropecuaria (INIA), Estación Experimental INIA Las Brujas, Ruta 48 Km 10, Canelones, Uruguay; 3 Instituto Nacional de Investigación Agropecuaria (INIA), Programa Cultivos de Secano, Estación Experimental La Estanzuela, Ruta 50 km 11, 70000 Colonia, Uruguay; 4 Laboratorio de Fisiología Vegetal, Centro de Investigaciones Nucleares, Facultad de Ciencias, Universidad de la República, Mataojo 2055, CP 11400 Montevideo, Uruguay

**Keywords:** *Diaporthe* pathogens, Soybean, Genomes, RNAseq, Pathogenicity factors, Secretome, Effectors

## Abstract

**Background:**

*Diaporthe caulivora* is a fungal pathogen causing stem canker in soybean worldwide. The generation of genomic and transcriptomic information of this ascomycete, together with a comparative genomic approach with other pathogens of this genus, will contribute to get insights into the molecular basis of pathogenicity strategies used by *D. caulivora* and other *Diaporthe* species.

**Results:**

In the present work, the nuclear genome of *D. caulivora* isolate (D57) was resolved, and a comprehensive annotation based on gene expression and genomic analysis is provided. *Diaporthe caulivora* D57 has an estimated size of 57,86 Mb and contains 18,385 predicted protein-coding genes, from which 1501 encode predicted secreted proteins. A large array of *D. caulivora* genes encoding secreted pathogenicity-related proteins was identified, including carbohydrate-active enzymes (CAZymes), necrosis-inducing proteins, oxidoreductases, proteases and effector candidates. Comparative genomics with other plant pathogenic *Diaporthe* species revealed a core secretome present in all *Diaporthe* species as well as *Diaporthe*-specific and *D. caulivora*-specific secreted proteins. Transcriptional profiling during early soybean infection stages showed differential expression of 2659 *D. caulivora* genes. Expression patterns of upregulated genes and gene ontology enrichment analysis revealed that host infection strategies depends on plant cell wall degradation and modification, detoxification of compounds, transporter activities and toxin production. Increased expression of effectors candidates suggests that *D. caulivora* pathogenicity also rely on plant defense evasion. A high proportion of the upregulated genes correspond to the core secretome and are represented in the pathogen-host interaction (PHI) database, which is consistent with their potential roles in pathogenic strategies of the genus *Diaporthe*.

**Conclusions:**

Our findings give novel and relevant insights into the molecular traits involved in pathogenicity of *D. caulivora* towards soybean plants. Some of these traits are in common with other *Diaporthe* pathogens with different host specificity, while others are species-specific. Our analyses also highlight the importance to have a deeper understanding of pathogenicity functions among *Diaporthe* pathogens and their interference with plant defense activation.

**Supplementary Information:**

The online version contains supplementary material available at 10.1186/s12864-022-08413-y.

## Background

Species of the genus *Diaporthe* and their asexual morph *Phomopsis* are endophytes, pathogenic, and saprophytic on a wide range of hosts worldwide [1–3]. Pathogenic *Diaporthe* species cause diseases on a wide range of plant hosts, including forest trees [4], citrus [5], pepper [6], sunflower [7], and soybean [8], among others. In soybean, *Diaporthe caulivora* (syn. *Diaporthe phaseolorum* var. *caulivora*) and *Diaporthe aspalathi* (syn. *Diaporthe phaseolorum* var. *meridionalis*) are the causal agents of soybean stem canker (SSC) [8–11]. *Diaporthe longicolla* also causes soybean stem canker, and it is usually found in association with *D. caulivora* and *D. aspalathi* [12, 13]. SSC causes important losses in soybean growing regions around the world [14–18], and disease symptoms are associated mainly to necrosis of the stem, with discoloration and leaf wilting [10, 13]. Currently, SSC management is performed by agronomic practices (crop rotation, avoidance of crop residue), fungicide applications, and the use of resistant cultivars [12, 19]. Host-plant resistance is currently the most effective strategy and therefore the identification of sources of resistance in soybean germplasms and breeding lines is of great importance. At present, five major genes that confer resistance to SSC caused by *D. aspalathi* and one for *D. caulivora* have been identified [10, 20, 21]. However, no commercial varieties with resistance to *D. caulivora* are available. Since *D. caulivora* is one of the principal causal agents of SSC in soybean producing countries [13, 19, 22], more information is needed to understand the interaction of this pathogen with the host plant in order to develop breeding strategies to control the disease.

Genomic information has allowed the identification of virulence factors and effector proteins in different pathogenic fungal species [23, 24], including plant cell wall degrading enzymes (PCWDEs) and enzymes involved in toxin production that are important for host colonization [25–27]. At present, 14 nuclear genome sequences of different *Diaporthe* species are available at NCBI [6, 26, 28–37]. However, genomic resources of the important pathogen *D. caulivora* are not available and transcriptomic profiling of *Diaporthe* species during host infection has not been performed. Genomic and transcriptomic approaches will give insights into the molecular basis of the pathogenicity strategies used by different *Diaporthe* species. In the present work, we report for the first time the genome sequence of *D. caulivora* and performed a comparative analysis with five available pathogenic *Diaporthe* genomes from different hosts [6, 13, 35–41]. Moreover, we present RNAseq data of *D. caulivora* during host plant infection. Our findings reveal the presence of pathogenicity factors that are shared between all *Diaporthe* species and *D. caulivora*-specific virulence components involved in host colonization and plant defense evasion.

## Results and discussion

### *Diaporthe caulivora* de novo genome sequencing, assembly and comparison with available *Diaporthe* genomes

The nuclear genome assembly of *D. caulivora* was resolved taking the advantages of PacBio sequences reads using FlyE v2.7. The assembly consisted in 10 contigs with a total length of 57,864,239 bp and a coverage of 270X (Table 1). The polishing step was performed using Minimap2 v2.18 by mapping the Pacbio raw reads back to the genome assembly [42], with a mapping rate of 97.8%. Genome assembly and the annotation was defined with high precision and completeness, determined by BUSCO analysis; 98% completeness in fungi_odb10.2019-11-20 database. We further looked for nuclear genomes of other *Diaporthe* species available at NCBI and selected five genomes, *D. capsici, D. citri, D. destruens*, *D. longicolla* and *D. phragmitis*, according to the quality of the assembly based on the total genome size, N50, number of contigs and number of N’s (Additional file 1, Table 1). Although *D. longicolla* has a lower quality assembly, it was included since this species has high occurrence in SCC lesions [13]. *D. capsici, D. citri, D. destruens* and *D. phragmitis* are pathogens with different host range (Additional file 2). Phylogenetic analysis with the internal transcribed spacer (ITS) and translation elongation factor 1-alpha gene (TEF1α) sequences of the six *Diaporthe* species, ex-type strains as well as other pathogenic *Diaporthe* species, confirmed the identity of the isolates used (Additional file 3). The genome assembly sizes of *D. capsici, D. destruens* and *D. phragmitis* ranged from 56,1 Mb to 58,3 Mb [6, 36, 37], and were comparable in size with *D. caulivora* genome (57,86 Mb), while *D. longicolla* and *D. citri* were ~ 7 Mb larger than the other species analyzed [26, 35] (Table 1).

**Table 1:** General features of *Diaporthe* genomes

***Diaporthe*** species	***D. caulivora***	***D. capsici***	***D. citri***	***D. destruens***	***D. longicolla***	***D. phragmitis***
Total genome size (bp)	57,864,239	57,558,510	63,685,968	56,108,228	64,714,568	58,328,132
Coverage (X)	270	ND	271	121	145	50
Number of contigs	10	20	34	47	985	28
Maximum contig length (bp)	14,464,108	8,755,198	12,370,252	6,293,594	1,124,325	7,711,659
N50 (bp)	8,708,519	5,171,887	5,472,022	2,479,481	204,364	3,550,333
GC content (%)	52.97	51.27	46.72	48.7	48.26	50.82
Complete BUSCO (%)	97.8	ND	98.5	97.93	98.21	97.9
Duplicated BUSCO (%)	0.6	ND	ND	3.33	ND	1
Fragmented BUSCO (%)	0.43	ND	ND	ND	ND	ND
Missing BUSCO (%)	1	ND	ND	ND	ND	1.1
Protein-coding gene number	18,385	14,425	15,921	13,754	16,606	12,393
Total gene length (bp)	23,421,216	23,205,508	26,007,773	ND	28,344,980	16,320,211
Average gene length (bp)	1690	1609	1633.5	ND	1709	1317
Protein-coding gene number (Augustus with *D. caulivora*)^a^	18,385	15,675	15,113	13,717	15,232	15,655
Predicted secretome ^a^	1501	1588	1383	1298	1535	1539
Predicted effectors > 80% probability^a^	1598	1204	1088	1103	1229	1168
Cytoplasmic effectors^a^	1448	1043	938	982	1073	1009
Apoplastic effectors^a^	150	161	150	121	156	159
Genbank accesion no.	BioProject PRJNA717308	WNXA00000000	JACTAD000000000	JACAAM000000000	JUJX00000000	JACDXY000000000
References	this paper	[6]	[34]	[36]	[28]	[37]

*ND* no data

^a^ data from this paper

Syntenic blocks analysis provides insights into genomic evolution between related species and defines regions of chromosomes between genomes that share a common order of homologous genes derived from a common ancestor [43]. A whole genome nucleotide alignment using MUMmer [44], implemented in SyMap V 5.0.6, allowed the identification of large regions in syntenic blocks between *D. caulivora* and the other five *Diaporthe* species with an average of 96% (Additional file 4). This analysis showed that *D. caulivora* genome has a range of 82 to 92 syntenic blocks with a coverage of 95% of the total length of the genomes respect to *D. capsici*, *D. citri*, *D. destruens* and *D. phragmitis* genomes. It is worth noting that *D. caulivora* contig 10 has a complete synteny with contig 19 of *D. capsici*. The other contigs of *D. caulivora* were highly conserved with a few rearrangements compared with the four *Diaporthe* species mentioned above. The high syntenic similarity among these five *Diaporthe* species indicates their close genetic relationship, evidenced by a strong overall conservation of genomic organization. The *D. longicolla* assembly was the only one obtained only by Illumina technology and showed the lowest values of synteny, which is consistent with the degree of fragmentation of the assembly.

### Gene prediction, functional annotation and orthologs

The annotation of the *D. caulivora* genome assembly was performed using a Fungap pipeline with combined abinitio strategies, homology-based searches and RNA-seq data, resulting in the annotation of 18,385 protein-coding regions. The functional annotation of predicted protein-coding genes was completed with the Blast2GO (B2Go) program [45]. The predicted genes were compared by BlastP against NCBI nr database (TaxId: *Fungi*) and classified by InterProScan v.5.19., Pfam protein families, InterPro domains, Gene Ontology Classification (GO), and metabolic pathways were recovered from proteins identified by BLAST using the B2Go annotation system (Additional file 5). Functional description could be assigned to 16,068 coding genes (87.4%). The remaining 2317 genes were searched at NCBI and 1787 of them did not show any hit and were considered as putative *D. caulivora*-specific genes validated by transcriptomic data (Additional file 5). Additional genome sequencing of more *Diaporthe* species will aid to confirm if these genes are only present in *D. caulivora* and not in other genomes of the genus. In order to make comparisons at gene levels with the five genomics assemblages of the other *Diaporthe* species included in this work, we performed gene prediction of the *Diaporthe* genomes with the Augustus web server (http://bioinf.uni-greifswald.de/webaugustus/), using *D. caulivora* protein-coding gene models as a training set. The total predicted coding genes were 15,675 genes models for *D. capsici*, 15,921 for *D. citri*, 13,754 for *D. destruens,* 16,606 for *D. longicolla*, and 12,393 for *D. phragmitis* (Table 1).

**Fig. 1:**
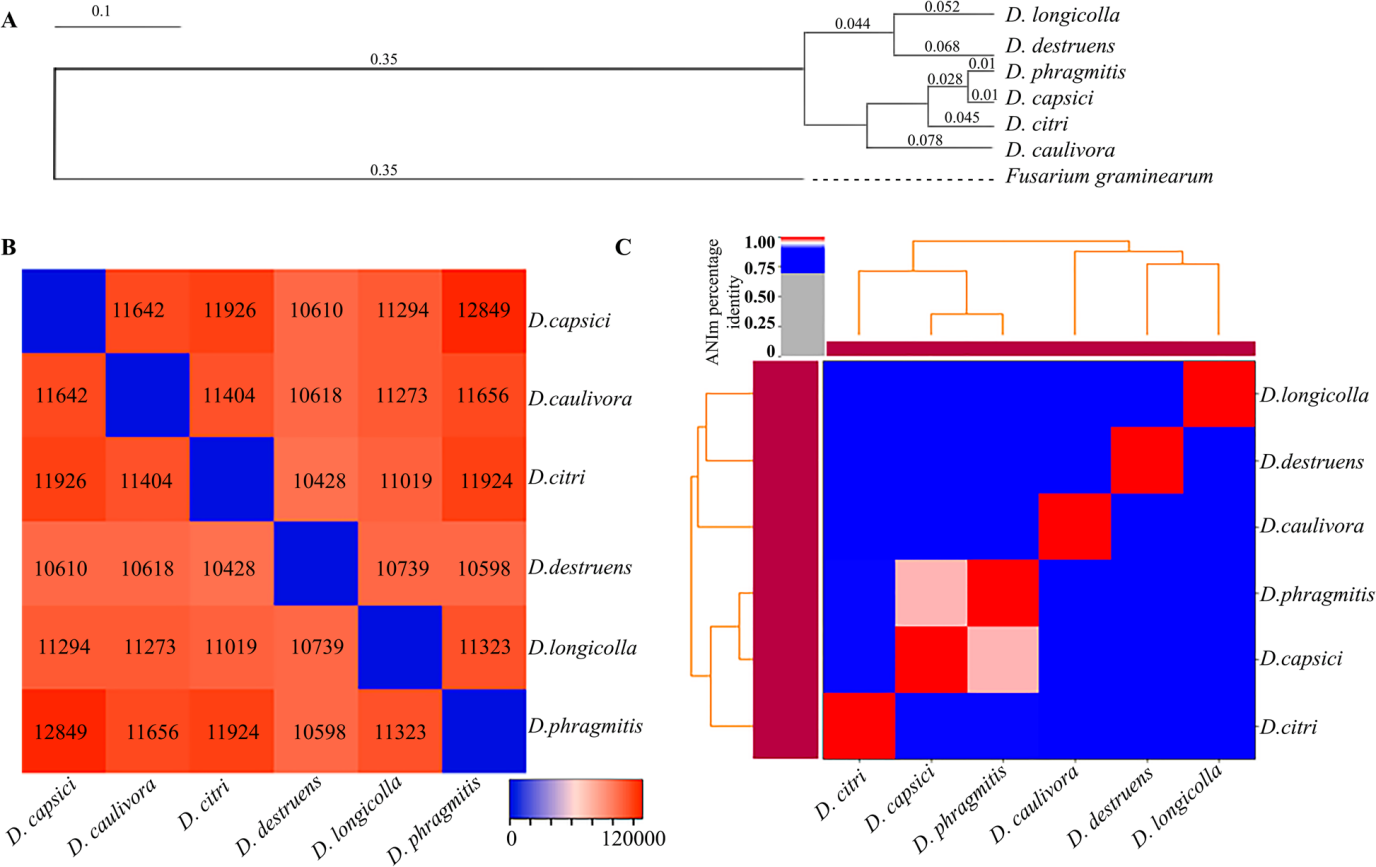
Orthologs and phylogenomic relationships of *D. caulivora* and other pathogenic *Diaporthe species*. **A** The species tree based on the orthologous groups was inferred by STAG and rooted by STRIDE. *Fusarium graminearum* was used as outgroup. **B** Heatmap showing the number of genes of each fungal species included in the orthogroups obtained by OrthoFinder. The scale represents the number of genes in orthogroups. **C** Heatmap of identity percentage amongs *Diaporthe* species based on the average nucleotide identity (ANI) analisys performed using PYANI v0.3. 0-alpha with MUMer to align the input sequences (ANIm). Scale in (**A**) represents divergence and substitutions per site

OrthoFinder analysis combined with an all-versus-all protein BLAST strategy was used to cluster protein orthologous groups and infer a phylogeny tree with the six *Diaporthe* species and *Fusarium graminearum* as outgroup (Fig. 1). The results show that an overall 94% (86–97%) of genes were assigned to orthologous groups shared by the six *Diaporthe* species. Phylogenetic analysis showed that the six *Diaporthe* species were divided into two main clusters (Fig. 1A). *Diaporthe caulivora* is phylogenetically distant from *D. capsici, D. citri* and *D. phragmitis,* which can be explained by the lower number of orthologous genes (87%) shared with these three species. *Diaporthe capsici, D. citri* and *D. phragmitis* are phylogenetically closely related, largely due to sharing nearly 98.,4% of the orthologous genes, which is in agreement with what has been shown by Gai et al. [35]. The second cluster included *D. destruens* and *D. longicolla.* Although *D. caulivora* and *D. longicolla* are soybean pathogens and they could be expected to be closely related, *D. longicolla* shares a similar number of genes assigned to orthologous groups with the other *Diaporthe* species regardless of the host specificity (Fig. 1B). Taking into account the quality of the analyzed assemblages, it is possible that specific genes of each species have not been fully recovered, unlike the *D. caulivora* genome presented in this work. Besides identifying the orthologous genes, we analyzed the average nucleotide identity (ANIm) of the species, which measure the nucleotide-genomic similarity between two genomes. The ANIm values between the different *Diaporthe* species varied from 78.6 to 96.4% and similar clusters as those obtained by orthologous analyses were obtained, except for *D. caulivora* which grouped together with *D. longicolla* and *D. destruens* (Fig. 1C).

### *Diaporthe caulivora*-specific and shared *Diaporthe* virulence components

**Fig. 2:**
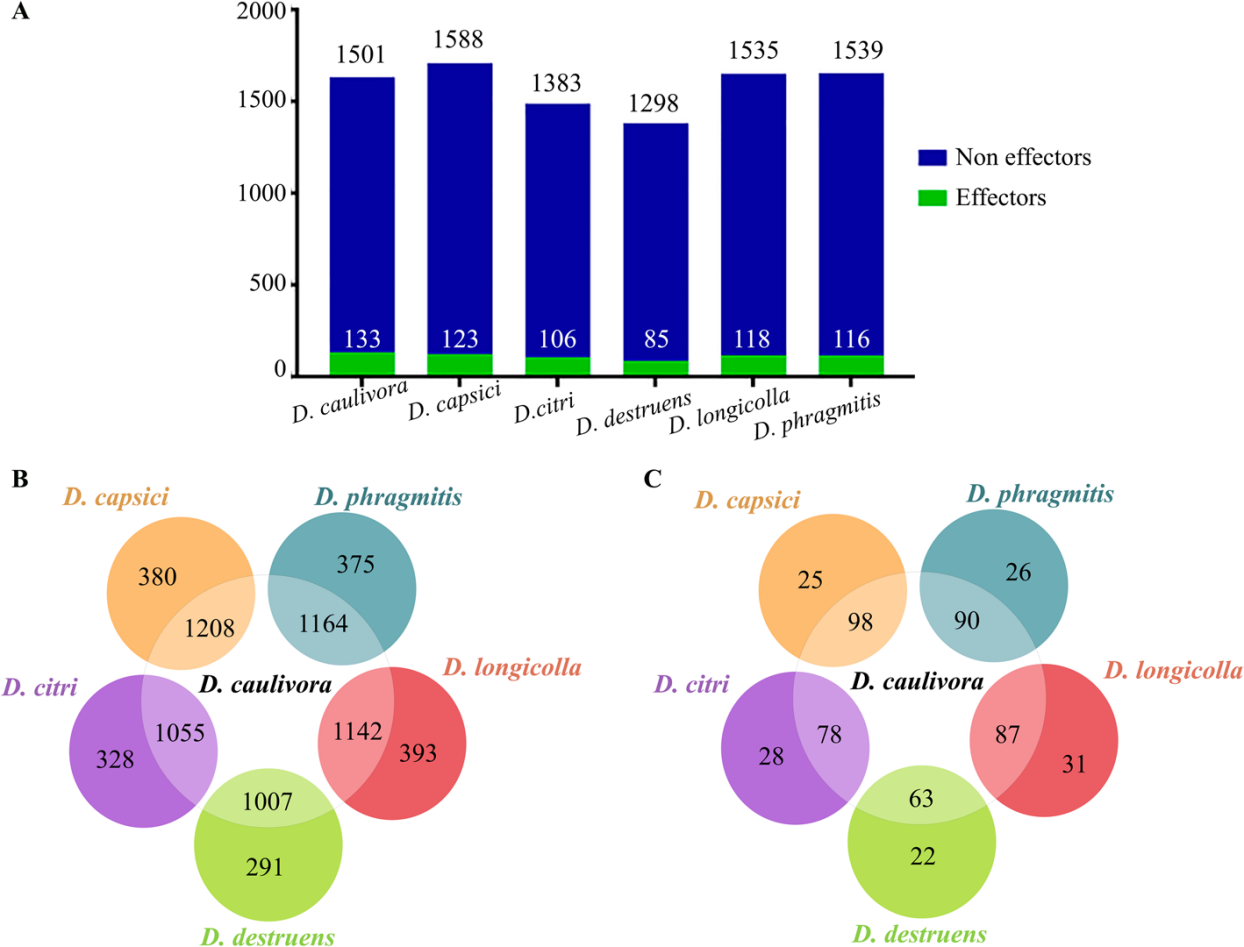
Number of predicted secreted proteins and effector candidates in *D. caulivora* and five other *Diaporthe* species. **A** Distribution of effectors in the different secretomes. Number of secreted proteins (**B**) and effector candidates (**C**) shared between *D. caulivora* and other *Diaporthe* species

Fungal secreted proteins mediate communication with the environment including host plants, and have important function in pathogenesis [46]. To define the set of proteins considered within the secretome of the six *Diaporthe* species analyzed in our work, we used SignalP v5.0, WoLF PSORT for Fungi and Phobius. *Diaporthe caulivora* has a total of 1501 genes encoding predicted secreted proteins. The predicted secretome of *D. capsici*, *D. longicolla* and *D. phragmitis* was similar in number (ranging from 1588 to 1535 proteins), while *D. citri* and *D. destruens* have a smaller secretome, represented by 1383 and 1298 proteins, respectively (Fig. 2A; Additional file 6). *Diaporthe caulivora* shared between 1007 and 1208 secreted proteins with the other five *Diaporthe* species (Fig. 2B).

**Fig. 3:**
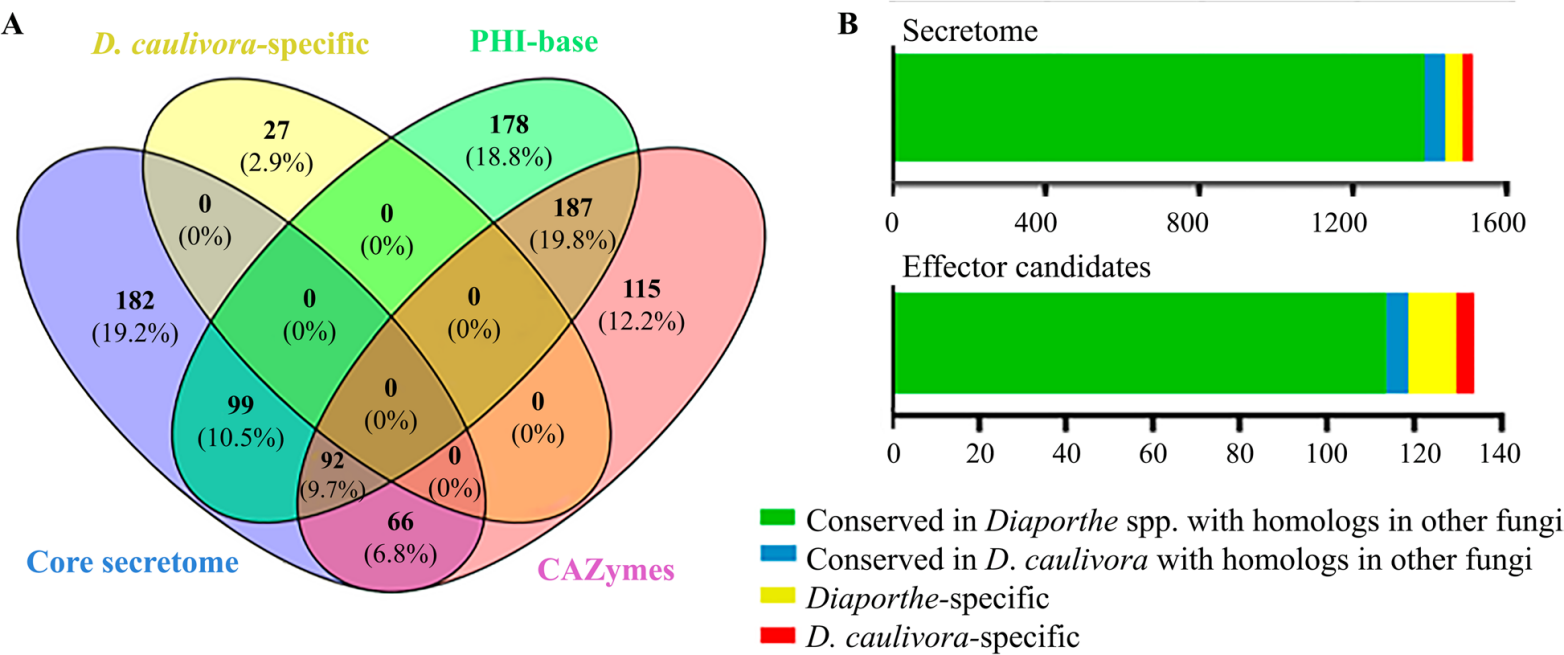
Shared and *D. cualivora*-specific secreted proteins and candidate effectors. **A** Venn diagram showing the overlap the core secretome, *D. caulivora*-specific secreted proteins, *D. caulivora* secreted CAZymes and PHI-base. **B** Conservation patterns of secreted proteins and effector candidates from *D. caulivora* indicating the number of conserved homologs in other *Diaporthe* species and fungi

In total 439 secreted proteins were in common between all *Diaporthe* species and were defined as the core secretome (Fig. 3A; Additional file 6). This core secretome includes virulence components related to plant cell wall degradation and modification, such as pectin and pectate lyases, glycoside hydrolases, carbohydrate esterases, endoglucanases and exoglucanases, a xylanase, as well as proteases, peptidases, lipases, peroxidases, among others. Combining the results of the six secretomes with a NCBI search that include recently sequenced *Diaporthe* and other fungal species, we identified 1375 *D. caulivora* predicted proteins that have conserved homologs in other *Diaporthe* and fungal pathogens (Fig. 3B, Additional file 7). The rest comprised 53 conserved proteins among *D. caulivora* and other fungal species, 46 *Diaporthe*-specific proteins and 27 putative *D. caulivora*-specific proteins that exhibited no hit with any other organism. Interestingly, five of the *Diaporthe*-specific proteins belong to the core secretome and represent relevant secreted proteins in the *Diaporthe* genus. *Diaporthe*-specific and *D. caulivora*-specific secreted proteins were all uncharacterized proteins whose roles in pathogenesis needs further investigation.

**Fig. 4:**
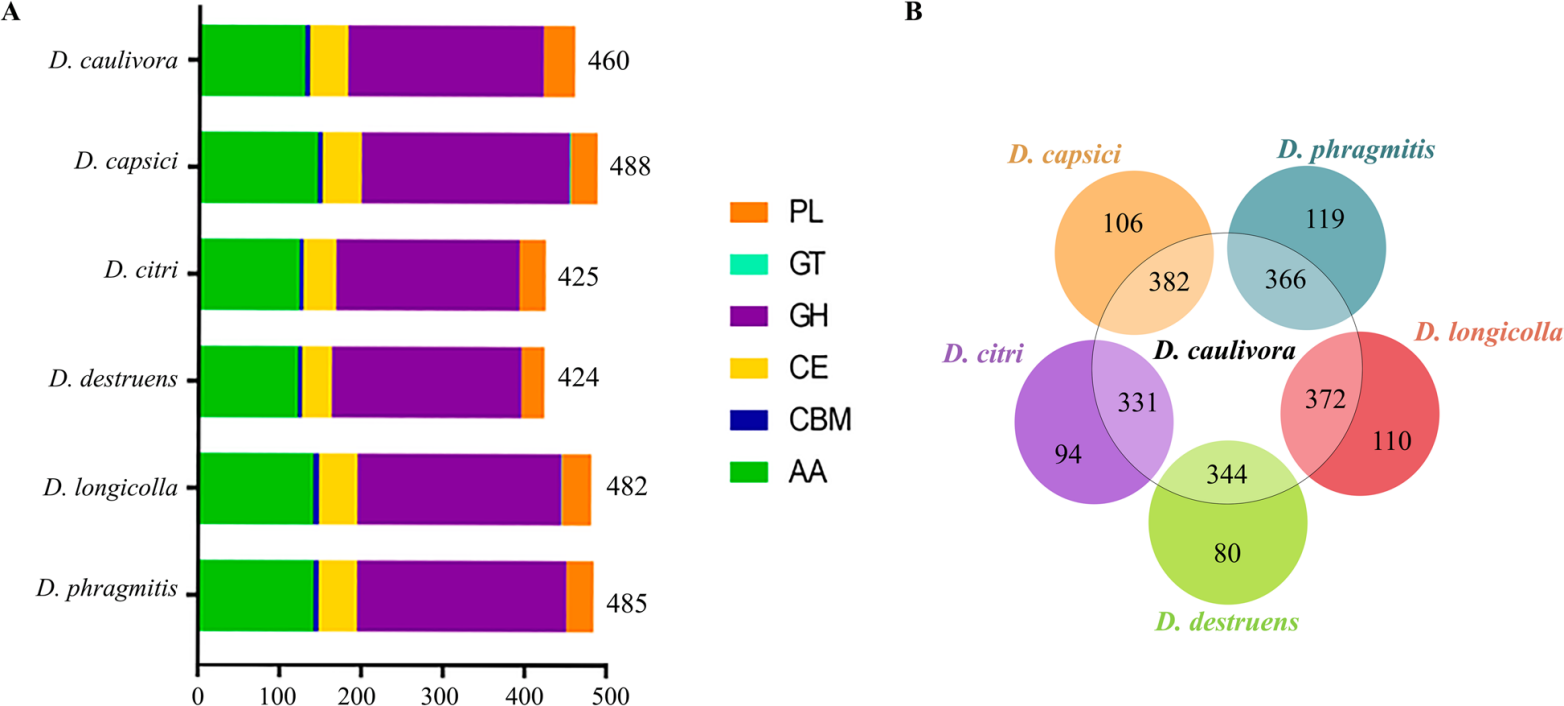
Comparative distribution of secreted CAZymes in different *Diaporthe* species. **A** Distribution according to classes of CAZyme module. PL, polysaccharide lyases; GT, glycosyl transferases; GH, glycoside hydrolases; CE, carbohydrate esterases; CBM, carbohydrate-binding module and AA, auxiliary activity. **B** Number of CAZymes shared between *D. caulivora* and other *Diaporthe* species

We looked into more detail to CAZYmes present in the *D. caulivora* secretome, and identified a total of 460 genes encoding CAZymes (Fig. 4; Additional file 8). The high proportion of CAZymes in the *D. caulivora* secretome (30.6%), is consistent with previous reports in other plant pathogens and *Diaporthe* species and emphasizes the importance to degrade efficiently polysaccharide materials present in the plant cell walls to facilitate infection and/or gain nutrition [26, 29, 47]. The other *Diaporthe* species have a similarly proportion of their secretome represented by CAZymes; 488 in *D. capsici* (30.7%), 425 in *D. citri* (30.7%), 424 in *D. destruens* (32.7%), 482 in *D. longicolla* (31.4%), and 485 in *D. phragmitis* (31.5%). The diversity of sub-categories of CAZymes, including Glycoside Hydrolase (GHs), Glycosyltransferase (GTs), Polysaccharide Lyase (PLs), Carbohydrate Esterase (CEs), were similar among the six *Diaporthe* species (Fig. 4A). The number of shared secreted CAZymes between *D. caulivora* and the other *Diaporthe* species varied between 331 and 382 (Fig. 4B), and 156 CAZymes were present in all species (Additional file 8). These findings suggest that secreted CAZymes play similar roles among *Diaporthe* species in terms of carbon utilization capabilities.

We further search in the Pathogen–Host Interaction (PHI)-base (Fig. 3A, Additional file 9), which catalogs experimentally verified pathogenicity, virulence and effector genes from different plant pathogens [48]. In total, 556 secreted proteins of *D. caulivora* (37%), were identified in the PHI-base and among them, 287 were related to reduced virulence or loss of pathogenicity mutant phenotypes, 18 to increased virulence and 35 to effectors (Additional file 9). From them, 191 belongs to the core secretome and half of them correspond to CAZymes reinforcing their important role in *Diaporthe* pathogenicity (Fig. 3A). Interestingly, several *D. caulivora* secreted proteins that are shared with other pathogenic fungi but were absent in available genomes of *Diaporthe* species include CAZymes, a kievitone hydratase involved in phytoalexin detoxification [49], several FAD-binding domain-containing proteins, a putative versicolorin B synthase involved in mycotoxin production [50], among others (Additional file 7). Five of these *D. caulivora* secreted proteins, represent homologs of fungal genes that were assigned as effectors or result in reduced virulence in knockout or mutant experiments, including two LysM domain-containing proteins, an endo-beta-1,6-glucanase, a pectin lyase-like protein, and a minor extracellular protease vpr (Additional file 9). Moreover, several genes encoding important virulence proteins in other fungi, such as necrosis- and ethylene-inducing proteins (NEP), transporters, oxidoreductases, proteases, hypersensitive response-inducing proteins, CFEM domain-containing protein were also present in the secretomes of several of the analyzed *Diaporthe* species, and most of them have PHI-base accessions hits.

Effectors are proteins or small molecules secreted by pathogens, which manipulate host cells, facilitating infection and interfering with host immunity [23, 51]. We identified 133 secreted *D. caulivora* effector candidates (Fig. 2A), including pectate and other polysaccharide lyases, glycoside hydrolases, a pathogenesis-related protein, a hypersensitive-inducing protein, peptidases, carbohydrate esterase and several hypothetical proteins (Additional file 10). The number of predicted effectors in the other five *Diaporthe* species ranged from 85 to 123 (Fig. 2A; Additional file 10). *Diaporthe caulivora* shared between 63 to 98 effectors candidates with the other five *Diaporthe* species (Fig. 2C). Nine effector candidates were considered core effectors since they were present in all *Diaporthe* species, including four CAZymes (pectate lyase, polysaccharide lyase, 1,4-beta-D-glucan cellobiohydrolase and xylanase), a protein CAP22 and four hypothetical proteins. Most of the *D. caulivora* effector candidates were also found in other *Diaporthe* species and other fungi, 11 were *Diaporthe*-specific and four were *D. caulivora*-specific (Fig. 3B, Additional file 7). All *Diaporthe*-specific and *D. caulivora*-specific effector candidates are hypothetical proteins, which lack a conserved domain. Moreover, of the total secreted *D. caulivora* effector candidates, only 17 were identified in the PHI-base; 8 were related to reduced virulence mutant phenotypes and four were identified as effectors (Additional file 9). These virulence factors include several CAZymes, comprising four CAZymes of the core effectors, and a sterigmatocystin biosynthesis peroxidase involved in toxin production [52]. Taken together, our results revealed that the genome of *D. caulivora* has a large array of pathogenicity-related genes, most of which are in common with other *Diaporthe* and fungal pathogens, while others are *Diaporthe*-specific or *D. caulivora*-specific. Further studies are needed to reveal the function of these genus- and species-specific effector candidates.

### *Diaporthe caulivora* genes encoding virulence factors and effector candidates are induced during soybean infection

**Fig. 5:**
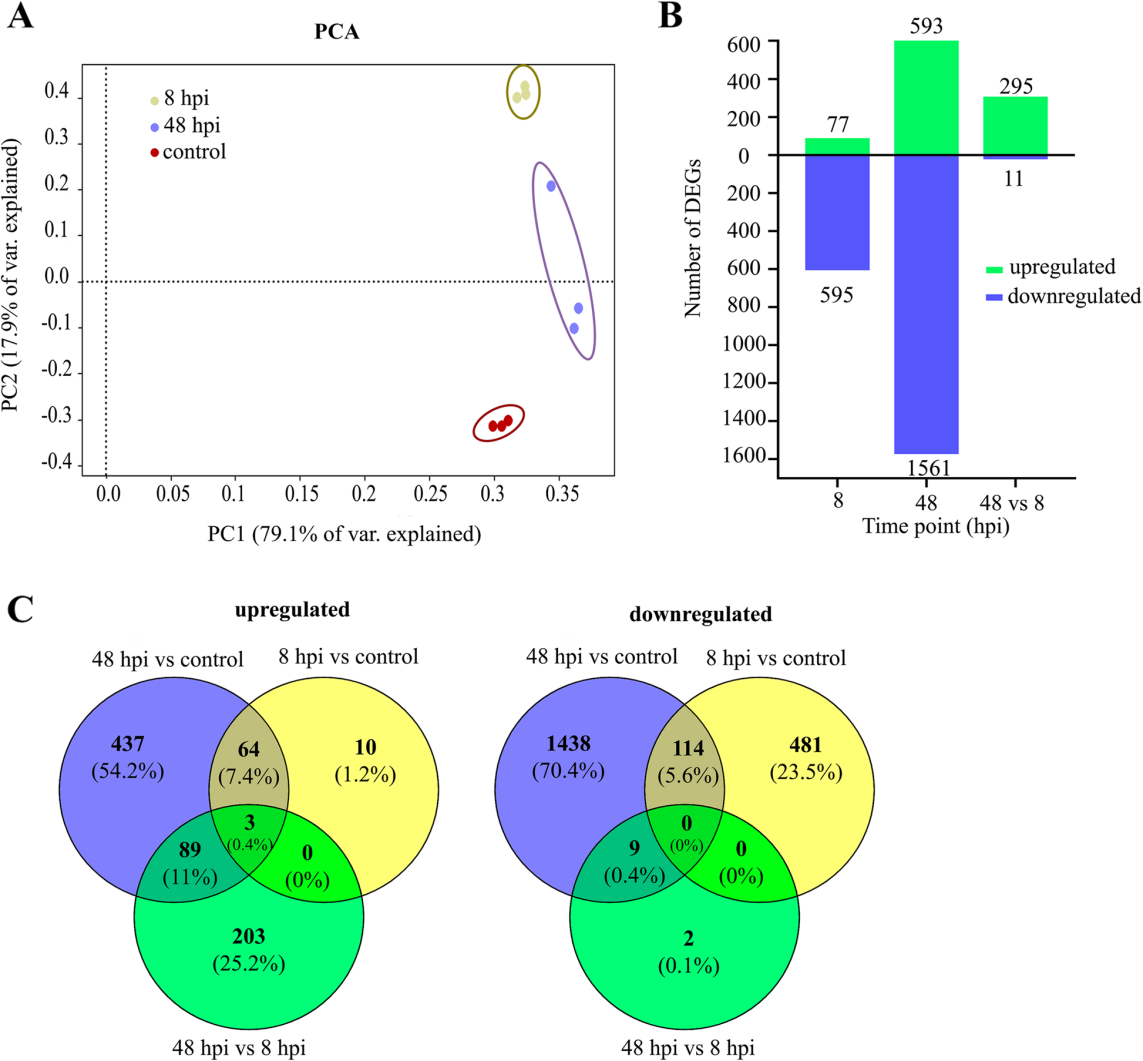
Differentially expressed *D. caulivora* genes during soybean infection. **A** Principal component analysis (PCA) of the transcriptomic data from RNA-seq. Colored dots denote each biological replicate. **B** Number of differentially expressed genes (DEGs). **C** Venn diagrams from *D. caulivora* comparisons at 8 hpi vs control, 48 hpi vs control and 48 vs. 8 hpi. Log2 FC ≥ 2.0 or ≤ − 2.0 and false discovery rate (FDR) ≤ 0.05 were considered for DEGs identification. In Venn diagrams, the overlap of expressed fungal genes can be observed

**Fig. 6:**
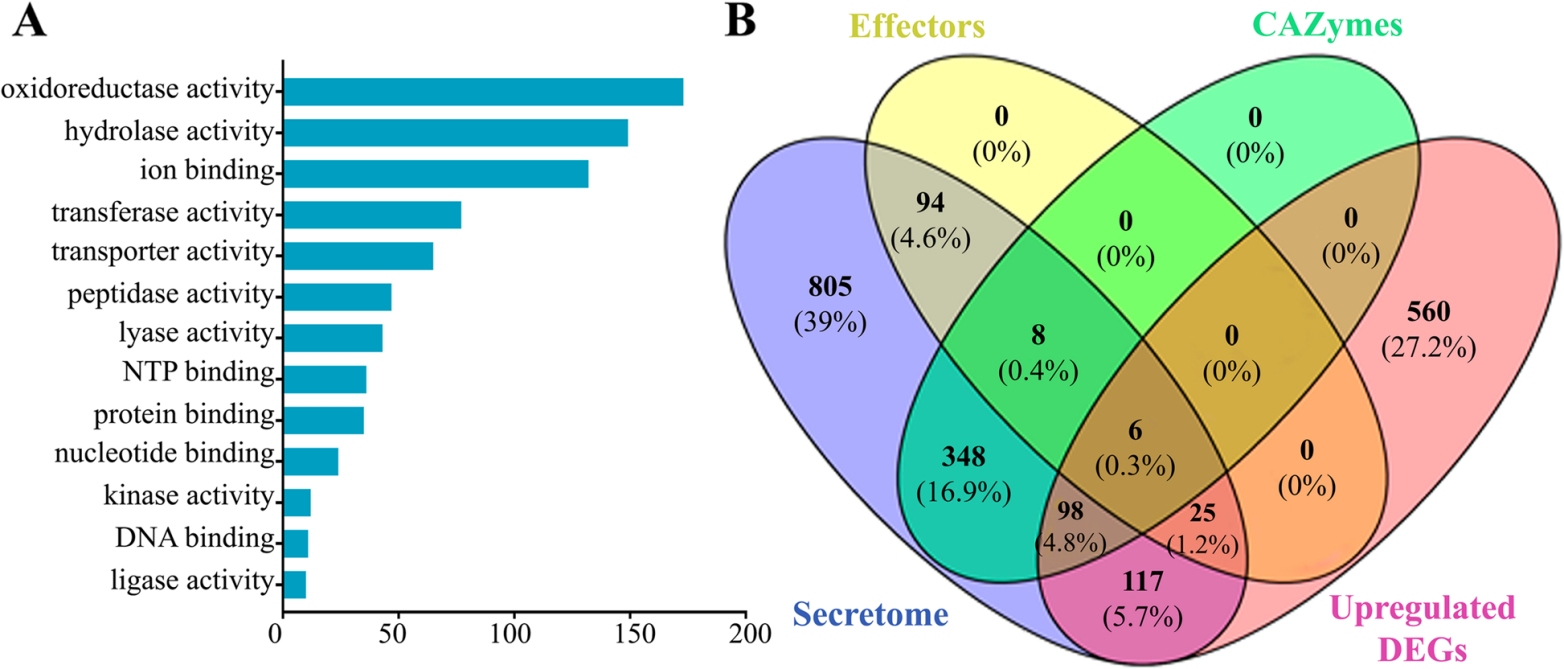
**A** Enriched Gene Ontology (GO) terms distribution for molecular function (MF) of upregulated differentially expressed genes (DEGs) in *D. caulivora* during soybean infection. **B** Venn diagram showing the overlap of upregulated DEGs and different gene categories relevant to *D. caulivora* pathogenicity

In order to identify *Diaporthe* genes involved in pathogenicity, we performed transcriptional profiling of two early stages of *D. caulivora* infection of soybean plants (8 and 48 hpi) and included *D. caulivora* mycelium grown on PDA medium (Additional file 11). A total of 69,940,418 reads mapped to the *D. caulivora* genome and were considered for further analyses. Biological variability within replicates was analyzed by principal component analysis (PCA). As shown in Fig. 5A, the first principal component (PC1) accounted for 79.1% of the total variation and separates the two time points (8 and 48 hpi), and the control *D. caulivora* samples. In total, 306 *D. caulivora* genes were differentially expressed in plant tissues (48 vs 8 hpi), 295 genes were upregulated and 11 downregulated (Fig. 5B; Additional file 12). In order to obtain more information on the infection process, we also compared differential expression between samples at 8 hpi and 48 hpi with mycelium samples grown on PDA. We identified 2635 additional *D. caulivora* differentially expressed genes (DEGs); 77 and 593 were upregulated, and 595 and 1561 were downregulated at 8 and 48 hpi, respectively (Fig. 5B-C; Additional file 12). We further focused on the total upregulated DEGs of the three comparisons (806 genes), since they could encode pathogenicity-related proteins involved in *D. caulivora* infection strategies. The functions of these DEGs were significantly enriched in several Molecular Function enriched GO terms, including oxidoreductase activity, hydrolase activity, lyase activity, ion binding and transporter activity (Fig. 6A; Additional file 12).

We identified a high proportion of DEGs showing homology to genes previously reported to be involved in fungal infection processes. During *D. caulivora* infection, genes encoding CAZymes, proteins involved in detoxification and transport of toxic compounds, proteases and effectors were upregulated (Additional file 12). A search against the PHI-base, predicted 119 upregulated *D. caulivora* genes that may be involved in pathogenicity (Fig. 6B, Additional file 13). Among them, 46 were related to reduced virulence or loss of pathogenicity mutant phenotypes and 15 to effectors, including CAZymes such as lectins, xyloglucan-specific endo-beta-1,4-glucanase, pectin and pectate lyases, as well as proteases, peptidases, among others (Additional file 13). Of the total upregulated DEGs, 246 encode proteins that were present in our predicted secretome (Fig. 6B). Interestingly, 106 (43%) of these upregulated secreted proteins belong to the core secretome, indicating their contribution to *Diaporthe* pathogenesis (Additional file 13). Moreover, of the 104 upregulated *D. caulivora* genes encoding secreted CAZymes (Fig. 6B), 47 were present in the core secretome (Additional file 12), including polygalacturonases, endoglucanases, exoglucanases, pectate lyases, pectin lyases, glycoside hydrolases, xyloglucan-specific endo-beta-1,4-glucanase, mannan endo-1,4-beta-mannosidase, rhamnogalacturonan acetylesterases, among others. The number of upregulated DEGs and expression levels of CAZymes-encoding genes, including PCWDEs, increased at 48 hpi compared to 8 hpi (Additional file 12), highlighting the important role played by these enzymes in soybean tissue breakdown and host penetration. In addition, seven genes encoding secreted proteins present only in *D. caulivora* and not in other *Diaporthe* species were upregulated, including a kievitone hydratase and six hypothetical proteins, three of which are *D. caulivora*-specific with no hit in public databases. Interestingly, kievitone hydratases are involved in detoxification processes of the phenylpropanoid kievitone, which play an important role in legumes defense against pathogen attack [53]. Of the total number of *D. caulivora* effectors, 23.3% (31 genes) were upregulated, including those encoding several CAZymes, a cell wall glycoprotein, several small secreted proteins, and hypothetical proteins (Additional files 12 and 13). Interestingly, five of the nine core effectors were upregulated during infection, including a xylanase, a polysaccharide lyase, CAP22, a putative 1,4-beta-D-glucan cellobiohydrolase and a hypothetical protein, indicating that they represent common effector candidates in different *Diaporthe* plant pathogens. Most of these common genes encode proteins involved in cell wall degradation and modification, and CAP22 is expressed in other pathogenic fungi in infection structures such as appresoria [54]. Moreover, one *Diaporthe*-specific (gene_10233.t1) and one *D. caulivora*-specific (gene_06736.t1) effector candidates were upregulated during infection.

Further inspection in transcriptomic data revealed possible strategies used by *D. caulivora*, and probably the other *Diaporthe* species, leading to host infection and tissues colonization. In addition to CAZymes-encoding genes, other upregulated genes belonging to the core secretome include proteins involved in pathogenesis such as peroxidases, SnodProt1, necrosis- and ethylene-inducing peptide 1 (Nep1)-like protein, saponin hydrolase, pathogenesis related protein, CAP22, peptidases, small secreted proteins and lipases. Upon entry into the host, fungal pathogens must resist toxic compounds produced by the plant [55]. Upregulation of a high number of genes encoding proteins involved in oxidoreductase processes during *D. caulivora* infection, like dehydrogenases, oxidases and reductases, is consistent with their important role played during fungal colonization and maintenance of redox status in both organisms [56] (Additional files 12 and 13). Several upregulated *D. caulivora* peroxidase-encoding genes belonging to the core secretome showed homology with genes included in the PHI-base. Like in other plant-pathogen interactions, *D. caulivora* and other *Diaporthe* peroxidases could be involved in lignin breakdown and detoxification of ROS produced by the host. In addition, expression of aldehyde dehydrogenases-encoding genes increased upon *D. caulivora* infection, which could be involved in pathogenicity through scavenging reactive aldehydes, fatty acid radicals, and other alcohol derivatives, as occur in other plant fungal pathogens [57] (Additional file 13). Moreover, 24 cytochrome P450 encoding genes were upregulated during *D. caulivora* infection, several of which exhibited functionally characterized homologs in PHI-base (Additional file 13). Since some cytochrome P450 are capable of detoxifying phytoalexins [58], and P450 monooxygenases are involved in the synthesis of some fungal toxins [59], they probably are also needed for *D. caulivora* pathogenesis. Interestingly, P450 families expanded significantly in several plant pathogens, including *D. ampelina* indicating their important roles during fungal pathogenesis [30]. Enzymes with oxidative-reductive properties also play relevant functions in degrading antimicrobial compounds such as phytoalexins and polyamines of plant origin [60]. Consistently, *D. caulivora* genes encoding enzymes involved in detoxification of plant defense molecules show high expression levels during early infection stages. Among them, we found several genes encoding putative pisatin demethylases, most of which were related to reduced virulence phenotypes in other pathosystems according to PHI-base (Additional file 13). In *Fusarium oxysporum* f. sp. *pisi*, a pisatin demethylase is responsible for detoxifying the pea phytoalexin pisatin [61]. Pisatin demethylases could encode glyceollin demethylases and detoxify soybean phytoalexin glyceollin [59]. Furthermore, two genes encoding dienelactone hydrolase (gene_04049.t1, gene_11950), involved in the β-ketoadipate pathway [62], were highly expressed, suggesting that they could be involved in detoxification mechanisms of plant defensive aromatic compounds. Interestingly, a saponin hydrolase present in the core secretome was upregulated during *D. caulivora* infection. These enzymes hydrolyze plant saponins that have antifungal activity and serve as potential chemical barriers against pathogens [63]. Thus, saponin hydrolases could be part of *Diaporthe* strategies to achieve successful host infection. On the other hand, a kievitone hydratase encoding gene (gene_03751.t1), which was only present in *D. caulivora* and other fungi such as *Fusarium oxysporum* and *Colletotrichum fructicola*, and not in other *Diaporthe* species, was upregulated during *D. caulivora* colonization. Kievitone hydratase is an important virulence factor in *Fusarium solani* that catalyzes the conversion of bean kievitone to a less toxic metabolite [49]. Furthermore, a gene encoding the phytotoxin SnodProt1, present in the core secretome, displays increased expression throughout the *D. caulivora* infection process. SnodProt1 proteins are involved in plant tissue colonization by several pathogenic fungi and produces ROS and necrosis of the plant tissues [64]. We have observed that *D. caulivora* infection induces ROS production in soybean stems (Mena, unpublished observations), and *D. aspalathi* elicitors activate the formation of nitric oxide (NO) in plant tissues that triggers the biosynthesis of antimicrobial flavonoids in soybean [65]. These findings, suggest that ROS and NO production play a role in plant-*Diaporthe* interactions.

Fungal toxins and necrosis inducing factors are produced to kill plant cells enabling nutrient uptake and mycelium growth. During *D. caulivora* infection, genes encoding secreted NEP (gene_04209.t1, gene_05426.t1 and gene_11576.t1) and a hypersensitive response-inducing protein (gene_18357.t1) were upregulated. Two of these NEP proteins belong to the core secretome, indicating their contribution to *Diaporthe* pathogenesis. Expression levels of genes involved in mycotoxin biosynthesis (gene_13234.t1, gene_10937.t1, gene_00256.t1, gene_05355.t1 and gene_15459.t1), and 11 genes encoding polyketide synthases (PKS) involved in toxin production in other fungi, increased during soybean infection (Additional file 12). Upregulation of this high number of PKS-encoding genes underline their involvement in *D. caulivora* pathogenesis, which is consistent with PHI-base hits with reduced virulence mutant phenotypes for PKS (gene_13009.t1, gene_01287.t1, gene_16446.t1) (Additional file 13), and previously reported reduced virulence of *D. helianthi* PKS1 mutant [27]. During sunflower infection, *D. helianthi* produces the polyketidic phytotoxin phomozin and related toxins, and purified phomozin causes disease symptoms [27]. Interestingly, the possible involvement of a phytotoxin produced by *D. caulivora* during SCC development has been previously reported [66].

Enzymes such as subtilases and alkaline proteinases have been shown to degrade plant defense proteins [67]. Increased expression of genes encoding three subtilisin-like proteinases (gene_11104.t1, gene_05325, gene_14820), an aspartic proteinase (gene_08152), and several other proteases in *D. caulivora*-infected tissues suggests their possible involvement in degradation of plant defense proteins. Consistently, disruption of genes with significant homology to gene_11104.t1 and gene_08152.t1, as well as other genes encoding proteases (gene_15501.t1, gene_15174.t1 and gene_06260.t1) showed reduced virulence in other plant pathogens (Additional file 13). The role of proteases and peptidases in *Diaporthe* pathogenesis is further supported by their presence in the core secretome.

Other upregulated genes encoding virulence factors include several transporters such as major facilitator superfamily (MSF) and ABC transporters, that in plant pathogenic fungi are responsible not only for export of compounds involved in pathogenesis, but also for excretion of plant-derived antimicrobial compounds [68]. In total, 6 MSF and four ABC transporters were upregulated during *D. caulivora* infection and showed significant homology to other transporters involved in virulence mechanisms in other plant pathogens (Additional files 12 and 13).

Taken together, our results suggest that early infection processes of soybean stems by *D. caulivora* depends on plant cell wall degradation and modification, detoxification of toxic compounds, transporter activities and toxin production. In addition, increased expression of genes encoding putative effector proteins during host colonization, some of which are species-specific, indicate that *D. caulivora* infection strategy also relies on plant defense evasion. Interestingly, several of these upregulated genes encoded proteins are part of the core secretome and represent common virulence components among *Diaporthe* species.

## Conclusions

Our findings give novel and relevant insights into the molecular players involved in pathogenicity of *D. caulivora* towards soybean plants, several of which are in common with other *Diaporthe* pathogens with different host specificity, while others are species-specific. Future studies towards understanding the role of effector candidates during the infection process of important pathogens among *Diaporthe* genus could reveal novel effector functions and plant targets that underpin *Diaporthe* pathogenic lifestyle. Our findings improve our understanding of *D. caulivora* pathogenicity mechanism involved in SCC development and allow genomic and transcriptomic comparisons among the most important pathogens belonging to the *Diaporthe* genus. Finally, the knowledge generated in this study provides a foundation for developing effective disease management strategies for SSC.

## Methods

All methods were performed in accordance with the relevant guidelines and regulations.

### *Diaporthe caulivora* inoculation

The *Diaporthe* virulent strain D57 was previously isolated from a stem canker lesion of a soybean plant grown in Uruguay and confirmed as *D. caulivora* by a phylogenetic tree generated from the analysis of the internal transcribed spacer (ITS) and translation elongation factor 1-alpha gene (TEF1α) [13]. *Diaporthe caulivora* D57 was growth in potato dextrose agar (PDA; Difco, Detroit, USA) at 24 °C in 12 h light/12 h darkness photoperiod. Plugs of a 5-day-old culture were used for plant inoculation. SSC-susceptible soybean (*Glycine max*) plants cultivar Williams (PI548631) were used for all assays. Seeds were obtained from USDA ARS Soybean germplasm collection (seed source 13 U-9280), and planted in 12-cm × 12-cm pots filled with a mix of soil and vermiculite at a rate of 3:1. Plants were grown at 24 °C under a 16 h light/8 h dark lighting regime. Soybean inoculation was performed on 3-week-old (V2 stage) plants, using the stem wounding method described by Mena et al. [13]. Briefly, wounding was performed by making a thin slice along the stem with a sterile scalpel (approx. 7 mm), 1 cm above the cotyledon, and single agar plugs bearing mycelium were carefully placed on the wound that was subsequently sealed with vaseline. PDA plugs without pathogen were placed on wounds as a control.

### Genomic DNA extraction, sequencing and de novo *D. caulivora* genome assembly

Agar plugs (5 mm in diameter) from the growing edge of 8-day-old cultures grown on PDA were transferred to new culture media and incubated at 24 °C under 12 h light and dark photoperiod. After 7 days, when the fungal growth completed the Petri dish, the mycelia were collected from three Petri dishes and frozen with liquid nitrogen. Approximately 150 mg of mycelium was ground with liquid nitrogen. Total genomic DNA was extracted using the DNeasy Plant Mini Kit (Qiagen, Hilden, Germany) according to the manufacturer’s instructions and quantified with Nanodrop 2000c (Thermo Scientific, Wilmington, USA). PacBio library preparation and sequencing were done in the Integrative Genomics Core Service, Beckman Research Institute, City of Hope (Monrovia, CA, USA). Briefly, a library for single-molecule real-time (SMRT) sequencing was constructed with an insert size of 15 kb using the SMRTbell™ Template Prep kit (Pacific Biosciences of CA, USA). The size of inserts was determined using the BluePippin device (Sage Science, MA, USA). Finally, the whole genome of *D. caulivora* was sequenced using the PacBio Sequel platform. Subreads were obtained using the SMRT Analysis RS. Flye v.2.7 [69] was used to assemble the data applying standard parameters and an estimated genome size of 50 Mb. The polishing and correction steps are included in the assembly pipelines. The genome graph structure was visualized with Bandage [70] to survey contiguity and ambiguities. Assembly statistics were obtained with QUAST v5.0.2 [71].

### Gene prediction and functional annotation

Gene prediction was performed using the FunGap pipeline [72]. Briefly, gene prediction was performed using three publicly available programs, Augustus [73], Braker 1 [74] and Maker [75] housing into the pipeline. Transcriptomic data of *D. caulivora* strain D57 obtained with the RNASeq protocol was processed with Trimmomatic v0.39 [76] and used for gene model prediction. The parameters using to run FunGAP were: –sister_proteome: Fusarium, − augustus_species fusarium_graminearum and transcript reads as –trans_read: paired-end. *Fusarium graminearum* was used as the reference species due to the relatively close phylogenetic relationship to *Diaporthe* among the genome sequences available in GenBank [26]. Single-copy fungal orthologs (fungi_odb10.2019-11-20 gene set) from Benchmarking Universal Single-Copy Orthologs (BUSCO v4) [77] were used to assess the completeness of the genome annotation.

Functional annotation was completed with Blast2GO [45], trough Omicbox software v. 2.0.36 (https://www.biobam.com/omicsbox). Gene models were compared with several databases (NCBI nonredundant protein database, Gene Ontology (GO) Consortium (http://geneontology.org), and InterpoScan) with BlastP finding single hit at an e-value threshold of 1E-20 using taxIds for fungi [78]. InterproScan analysis was used to identify domains in the *D. caulivora* genome [79]. Classification into GO categories was performed with Blast2GO software using *Fusarium graminearum* as a reference since protein domain information for *D. caulivora* was not available [80].

### Comparative genome analyses and phylogenetic analysis

The genomes of *D. capsici* strain GY-Z16 (Gene Bank accession number: WNXA00000000); *D. citri* strain NFHF_8_4 (Gene Bank accession number: JACTAD000000000); *D. destruens* strain CRI305_2 (Gene Bank accession number: JACAAM000000000); *D. longicolla* strain TWHP_74 (Gene Bank accession number: JUJX00000000); *D. phragmitis* strain NJD1 (Gene Bank accession number: JACDXY000000000) were downloaded from GenBank database of the NCBI website ((https://www.ncbi.nlm.nih.gov/nuccore).

ITS and TEF1α sequences were retrieved from the six *Diaporthe* genomes and aligned with MUSCLE (v3.8.31) [81], and configured for highest accuracy (MUSCLE with default settings). Iqtree software [82] was used to build a phylogenetic tree, with substitution model TIM2 + F + G4 estimated with Model finder [83] and ultraboostrap 1000.

Gene prediction for each *Diaporthe* assemblies downloaded was performed using AUGUSTUS web Server (http://bioinf.uni-greifswald.de/webaugustus/), with *D. caulivora* gene models from this work as a training set with default parameters (UTR prediction: false; report genes on: both strands; alternative transcripts: medium; allowed gene structure: predict any number of (possibly partial) genes.). The average nucleotide identity (ANI) of all *Diaporthe* species was calculated using PYANI v0.3. 0-alpha with MUMer to align the input sequences (ANIm) (https://github.com/widdowquinn/pyani). The orthologous analysis was conducted using OrthoFinder v2.5.4 [84] with all-versus-all BLAST strategy to define the orthogroups among the six *Diaporthe* species. Phylogenetic species rooted tree was inferred by multiple sequence alignment and maximum likelihood (options: “-S blast –M msa”) using all orthogroups from the six *Diaporthe* species based on Species Tree Inference from All Genes method (STAG).

Multiple software tools were jointly used to predict secreted proteins of the six *Diaporthe* species. SignalP 5.0 (http://www.cbs.dtu.dk/services/SignalP/) [85], WoLF PSORT for Fungi (http://www.genscript.com/psort/wolf_psort.html) [86], and Phobius (http://phobius.sbc.su.se/) [87], were employed to identify signal peptide signatures. Default parameters were used for all programs. For WoLF PSORT, proteins were considered if their extracellular score was ≥17 [88]. Predicted proteins to be signal-peptide positive by all three programs were selected and two additional filtering steps were applied to this set of sequences. First, putative membrane proteins were identified with TMHMM v2.0 (http://www.cbs.dtu.dk/services/TMHMM/) [89] and proteins with a number of expected transmembrane amino acids ≥18 were removed. Then, putative endoplasmic reticulum (ER) targeting proteins, predicted by using PS-Scan (http://prosite.expasy.org/scanprosite/) [90] with Prosite accession PS00014 were also removed. The remaining proteins were considered as secreted proteins and used in subsequent analyses. Additionally, EffectorP V3.0 was used to predict fungal effectors of all *Diaporthe* species [91–93].

The carbohydrate-active enzymes (CAZymes) were identified with dbCAN 5.0, which searches CAZy family-specific HMMs with HMMER3, and NCBI’s conserved domain database CDD [94]. Putative polyketide synthases (PKS) genes were identified using InterProScan and identification of conserved domain as indicated in [27]. To identify proteins involved in pathogenicity, the predicted secretome was used as a query for BlastP (e-value 1E-05) search against the pathogen-host interaction database (PHI-base v4.10) that catalogues experimentally verified pathogenicity, virulence and effector genes from fungal, oomycete and bacterial pathogens [48].

### RNA extraction, RNA sequencing and data processing

For transcriptomic analysis, samples were taken at 8 h post inoculation (hpi) and 48 hpi in soybean plants and *D. caulivora* mycelium grown on PDA plates for 7 days was used as a control. Each treatment consisted of three pots with three plants each at each infection time point, and three plates of mycelium grown on PDA plates. Soybean tissues (stem section of 1.5 cm including the wounded area) and *D. caulivora* mycelium were harvested for RNA extraction, immediately frozen in liquid nitrogen, and stored at − 80 °C. Soybean stem tissue samples (*n* = 3) were ground in liquid nitrogen. Total RNA from 100 mg of tissue was extracted and purified with TRIzol reagent (Invitrogen, USA), and using the Invitrogen PureLink RNA Extraction Mini kit (Invitrogen, USA), followed by a DNase I treatment (RNase-Free DNase I). The extraction was performed according to the manufacturer’s instructions. Quality of the isolated RNA was checked by running samples on 1.2% formaldehyde agarose gel. RNA concentration was measured using a NanoDrop 2000c (Thermo Scientific, Wilmington, USA). RNA quality control, library preparation, and sequencing were performed at Macrogen Inc. (Seoul, Korea). Three biological replicates are included by treatment. Libraries for each biological replicate were prepared for paired-end sequencing by TruSeq Stranded Total RNA LT Sample Prep Kit (Plant) with 1 μg input RNA, following the TruSeq Stranded Total RNA Sample Prep Guide, Part # 15,031,048 Rev. E. Sequencing was performed on Illumina platform (Illumina, CA, USA) by Macrogen Inc. (Seoul, Korea) to generate paired-end 101 bp reads, obtaining 41.6 to 65.2 M reads per sample with Q20 > 98% and Q30 > 95%. RNA-seq processing steps were done through Galaxy platform (https://usegalaxy.org/). Raw reads quality was subjected to a quality control check using FastQC software ver. 0.11.2 (http://www.bioinformatics.babraham.ac.uk/projects/fastqc/). Sequences were trimmed, and the adapters removed using Trimmomatic Version 0.38.0 software [76]. Additionally, to the default options, the following parameters were adjusted: adapter sequence TruSeq3 (paired-ended (PE), for MiSeq and HiSeq), always keep both reads of PE, and SLIDINGWINDOW: 4:15 HEADCROP: 13 MINLEN:50.Trimmed reads were mapped to the assembly of the *D. caulivora* genome obtained before using Hisat2 software [95]. The BAM files were obtained with Samtools View software v. 1.9 and then sorted by name with Samtools Sort software v. 2.0.3 [96], for further analysis.

Reads were counted using FeatureCounts software v. 1.6.4 [97]. Additionally to default options, parameters were adjusted for: count fragments instead of reads, allow read to map to multiple features, and use reference sequence file obtained before for *D. caulivora* annotation. Cluster analysis of replicates from each time point and control samples were performed by Principal Component Analysis (PCA) using pcaExplorer 2.16.0 software [98]. Counts were normalized to counts per million (cpm) using the TMM method and low expressed genes were filtered out applying a cpm value ≥3 in all samples. Differential expression analyses were performed using EdgeR software ver. 3.24.1 [99]. A false discovery rate (FDR) ≤ 0.05 was used to determine significant differentially expressed genes (DEGs) between *D. caulivora* grown on soybean stem and *D. caulivora* grown on PDA (control), and minimum log2 Fold Change 2 expression values were considered. Venn diagram drawing was performed with Venny [100].

## Supplementary Information

**Additional file 1. **General features of *Diaporthe* genomes.

**Additional file 2. **Characteristics of selected *Diaporthe* species.

**Additional file 3. **Phylogenetic tree generated from the analysis of internal transcribed spacer (ITS) and translation elongation factor 1-alpha gene (TEF1α) regions of the six *Diaporthe* species used in this study. The number at the branch nodes indicates bootstrap values (%) built on 1000 replications. The *Diaporthe* species used in this study are indicated in red and the ex-types strains in black. *Fusarium graminearum* was used as outgroup. ITS and TEF1α sequences of *D. destruens* were only obtained from the genome of strain CRI305_2 since sequences of other *D. destruens* strains were not available at NCBI.

**Additional file 4. **Synteny analysis between *D. caulivora* and other *Diaporthe* species. The synteny circle plots show the large synthenic blocks between *D. caulivora* and the other five *Diaporthe* species obtained using SyMAP.

**Additional file 5. **Gene prediction and annotation of *D. caulivora*.

**Additional file 6. ***Diaporthe* genes encoding predicted secreted proteins shared with one or more *Diaporthe* species or present only in one species.

**Additional file 7. **Predicted *D. caulivora* secreted proteins and effector candidates absent in the other five *Diaporthe* genomes. A NCBI search was performed showing hit with other *Diaporthe* and fungal species.

**Additional file 8. **Predicted CAZymes in the different *Diaporthe* species. CAZymes shared with one or more *Diaporthe* species or present only in one species are indicated.

**Additional file 9. **Summary of *D. caulivora* genes encoding predicted secreted proteins and effector candidates with functionally characterized homologs in Pathogen-Host Interaction database (PHI-base).

**Additional file 10. **Effectors candidates in *Diaporthe* secretomes shared with one or more *Diaporthe* species or present only in one species.

**Additional file 11. **Summary of mapped reads of *D. caulivora* in the RNA-Seq libraries. 1–3 indicate the three biological replicates during soybean infection at the indicated time points and control PDA grown mycelium.

**Additional file 12. **List of *D. caulivora* differentially expressed genes (DEGs) during soybean infection at 8 and 48 hpi.

**Additional file 13. **List of pathogenicity related *D. caulivora* differentially expressed genes (DEGs) present in different categories: secretome, CAZymes, effectors, and their functionally characterized homologs in PHI-base.

## Data Availability

The nuclear assembly data of *D. caulivora* (D57) was deposited into NCBI’s Genome database (https://www.ncbi.nlm.nih.gov/genome/) under the BioProject ID: PRJNA717308. All raw RNA-Seq read data were deposited at NCBI Short Read Archive (http://www.ncbi.nlm.nih.gov/sra/) under the BioProject ID PRJNA717275. The data are under embargo until publication.

## Abbreviations

CAZymes: Carbohydrate-active enzymes
DEG: Differentially expressed gene
GO: Gene Ontology
PCA: Principal Component Analysis
PDA: Potato dextrose agar
PCWDEs: Plant cell wall degrading enzymes SSC: soybean stem canker
PHI-base: Pathogen–Host Interaction base
ROS: Reactive Oxygen Species

## References

[1] Liu JK, Hyde KD, Jones EBG, Ariyawansa HA, Bhat DJ, Boonmee S (2015). Fungal diversity notes 1-110: taxonomic and phylogenetic contributions to fungal species. Fungal Divers.

[2] Hyde KD, Hongsanan S, Jeewon R, Bhat DJ, McKenzie EHC, Jones EBG (2016). Fungal diversity notes 367-491: taxonomic and phylogenetic contributions to fungal taxa. Fungal Divers.

[3] Marin-Felix Y, Hernández-Restrepo M, Wingfield MJ, Akulov A, Carnegie AJ, Cheewangkoon R (2019). Genera of phytopathogenic fungi: GOPHY 2. Stud Mycol.

[4] Yang Q, Fan XL, Guarnaccia V, Tian CM (2018). High diversity of *Diaporthe* species associated with twelve new species described. MycoKeys.

[5] Udayanga D, Castlebury LA, Rossman AY, Hyde KD (2014). Species limits in *Diaporthe*: molecular re-assessment of *D citri D cytosporella D foeniculina* and *D rudis*. Persoonia.

[6] Fang X, Qin K, Li S, Han S, Zhu T, Fang X, Qin K (2020). Whole genome sequence of *Diaporthe capsici* a new pathogen of walnut blight. Genomics.

[7] Thompson SM, Tan YP, Young AJ, Neate SM, Aitken EAB, Shivas RG (2011). Stem cankers on sunflower (*Helianthus annuus*) in Australia reveal a complex of pathogenic *Diaporthe* (*Phomopsis*) species. Persoonia.

[8] Udayanga D, Castlebury LA, Rossman AY, Chukeatirote E, Hyde KD (2015). The *Diaporthe sojae* species complex: phylogenetic re-assessment of pathogens associated with soybean cucurbits and other field crops. Fungal Biol.

[9] Fernández FA, Philips DV, Russin JS, Rupe JC (1999). *Diaporthe-Phomopsis* complex. Compendium of soybean diseases Hartman GLJB Sinclair and JC Rupe (4th ed).

[10] Pioli RN, Morandi EN, Martínez MC, Lucca F, Tozzini A, Bisaro V, Hopp HE (2003). Morphologic molecular and pathogenic characterization of *Diaporthe phaseolorum* variability in the core soybean-producing area of Argentina. Phytopathology.

[11] Santos JM, Vrandečić K, Cosić J, Duvnjak T, Phillips AJ (2011). Resolving the Diaporthe species occurring on soybean in Croatia. Persoonia.

[12] Ghimire K, Petrović K, Kontz BJ, Bradley CA, Chilvers MI, Mueller DS, et al. Inoculation method impacts symptom development associated with *Diaporthe aspalathi D caulivora* and *D longicolla* on soybean (*Glycine max*). Plant Dis. 2019;103(4):PDIS06181078RE. 10.1094/PDIS-06-18-1078-RE 30742552

[13] Mena E, Stewart S, Montesano M, Ponce de León I (2020). Soybean stem canker caused by *Diaporthe caulivora*; pathogen diversity colonization process and plant defense activation. Front Plant Sci.

[14] Backman PA, Weaver DB, Morgan-Jones G (1985). Soybean stem canker: an emerging disease problem. Plant Dis.

[15] Grijalba PE, Guillin E (2007). Occurrence of soybean stem canker caused by *Diaporthe phaseolorum* var *caulivora* in the southern part of Buenos Aires province Argentina. Australas Plant Dis Notes.

[16] Wrather JA, Koenning SR. Effects of diseases on soybean yields in the United States 1996 to 2007. Plant Health Prog. 2009;10(1).

[17] Maldonado dos Santos JV, Ferreira EGC, Passianotto AL, Brumer BB, Santos ABD, Soares RM (2019). Association mapping of a locus that confers southern stem canker resistance in soybean and SNP marker development. BMC Genomics.

[18] Bandara AY, Weerasooriya DK, Bradley CA, Allen TW, Esker PD (2020). Dissecting the economic impact of soybean diseases in the United States over two decades. PLoS One.

[19] Roth MG, Webster RW, Mueller DS, Chilvers MI, Faske TR, Mathew FM (2020). Integrated management of important soybean pathogens of the United States in changing climate. J Integr Pest Manag.

[20] Chiesa MA, Pioli RN, Morandia EN (2009). Specific resistance to soybean stem canker conferred by the Rdm4 locus. Plant Pathol.

[21] Peruzzo AM, Hernández FE, Pratta GR, Ploper LD, Pioli RN (2019). Identification and inheritance of an *Rdc* gene resistance to soybean stem canker (*Diaporthe phaseolorum* var *caulivora*). Eur J Plant Pathol.

[22] Grijalba P, Ridao AC (2012). Survival *of Diaporthe phaseolorum* var *caulivora* (causal agent of soybean stem canker) artificially inoculated in different crop residues. Trop Plant Pathol.

[23] Selin C, de Kievit TR, Belmonte MF, Fernando WGD (2016). Elucidating the role of effectors in plant-fungal interactions: Progress and challenges. Front Microbiol.

[24] Klosterman SJ, Rollins JR, Sudarshana MR, Vinatzer BA (2016). Disease management in the genomics era - summaries of focus issue papers. Phytopathology.

[25] Chang HX, Yendrek CR, Caetano-Anolles G, Hartman GL (2016). Genomic characterization of plant cell wall degrading enzymes and in silico analysis of xylanses and polygalacturonases of *Fusarium virguliforme*. BMC Microbiol.

[26] Li S, Darwish O, Alkharouf NW, Musungu B, Matthews BF (2017). Analysis of the genome sequence of *Phomopsis longicolla*: a fungal pathogen causing *Phomopsis* seed decay in soybean. BMC Genomics.

[27] Ruocco M, Baroncelli R, Cacciola SO, Pane C, Monti MM, Firrao G (2018). Polyketide synthases of *Diaporthe helianthi* and involvement of DhPKS1 in virulence on sunflower. BMC Genomics.

[28] Li S, Song Q, Pingsheng J, Cregan P (2015). Draft genome sequence of *Phomopsis longicolla* type strain TWH P74 a fungus causing phomopsis seed decay in soybean. Genome Announc..

[29] Li S, Song Q, Martins AM, Cregan P (2016). Draft genome sequence of *Diaporthe aspalathi* isolate MS-SSC91 a fungus causing stem canker in soybean. Genomics data.

[30] Morales-Cruz A, Amrine KC, Blanco-Ulate B, Lawrence DP, Travadon R, Rolshausen PE (2015). Distinctive expansion of gene families associated with plant cell wall degradation secondary metabolism and nutrient uptake in the genomes of grapevine trunk pathogens. BMC Genomics.

[31] Baroncelli R, Scala F, Vergara M, Thon M, Ruocco M. Draft whole-genome sequence of the *Diaporthe helianthi* 7/96 strain causal agent of sunflower stem canker. Genom Data. 2016;10. 10.1016/jgdata20161100. 10.1016/j.gdata.2016.11.005 PMC5110471 27872817

[32] Savitha J, Bhargavi SD, Praveen VK (2016). Complete genome sequence of the endophytic fungus *Diaporthe (Phomopsis) ampelina*. Genome Announc.

[33] Tulsook K, Isarangkul D, Sriubolmas N, Kittakopp P, Wiyakrutta S (2020). Genome sequence of *Diaporthe* sp strain HANT25 an endophytic fungus producing mycoepoxydiene. Microbiol Resour Announc.

[34] Liu X, Chaisiri C, Lin Y, Yin W, Luo C (2021). Whole-genome sequence of *Diaporthe citri* isolate NFHF-8-4 the causal agent of citrus melanose. Mol Plant-Microbe Interact.

[35] Gai Y, Xiong T, Xiao X, Li P, Zeng Y, Li L, Riely BK, Li H (2021). The genome sequence of the *Citrus* melanose pathogen *Diaporthe citri* and two citrus-related *Diaporthe* species. Phytopathology.

[36] Huang L, Zhang X, Yang Y, Zou H, Fang B, Liu W (2021). High-quality genome resource of *Diaporthe destruens* causing foot rot disease of sweet potato. Plant Dis.

[37] Wang X, Dong H, Lan J, Liu Y, Liang K, Lu Q (2021). High-quality genome resource of the pathogen of *Diaporthe* (*Phomopsis*) *phragmitis* causing kiwifruit soft rot. Mol Plant-Microbe Interact.

[38] Hosseini B, El-Hassan A, Immanuel H, Voegele R (2020). Analysis of the species spectrum of the *Diaporthe/Phomopsis* complex in European soybean seeds. Mycol Prog.

[39] Zambelli A, Mancebo MF, Bazzalo ME, Reid RJ, Sanchez MC, Kontz BJ, Mathew FM (2021). Six species of *Diaporthe* associated with *Phomopsis* stem canker of sunflower in southern pampean region of Argentina. Plant Health Prog.

[40] Nozaki M, Camargo M, Barreto M. Caracterização de *Diaporthe citri* em diferentes meios de cultura condições de temperatura e luminosidade. Fitopatol Bras. 2004;29(4):429–32.

[41] Mathew FM, Rashid KY, Gulya TJ, Markell SG (2015). First report of *Phomopsis* stem canker of sunflower (*Helianthus annuus*) caused by *Diaporthe gulyae* in Canada. Plant Dis.

[42] Li H (2018). Minimap2: pairwise alignment for nucleotide sequences. Bioinformatics.

[43] Tang H, Lyons E, Pedersen B, Schnable JC, Paterson AH, Freeling M. Screening synteny blocks in pairwise genome comparisons through integer programming. BMC Bioinformatics. 2011;102:1–11. 10.1186/1471-2105-12-102 PMC3088904 21501495

[44] Kurtz S, Phillippy A, Delcher AL, Smoot M, Shumway M, Antonescu C, Salzberg SL (2004). Versatile and open software for comparing large genomes. Genome Biol.

[45] Conesa A, Götz S. Blast2gGO: A comprehensive suite for functional analysis in plant genomics. Int J Plant Genom. 2008;2008:619832. 10.1155/2008/619832 PMC2375974 18483572

[46] McCotter SW, Horianopoulos LC, Kronstad JW (2016). Regulation of the fungal secretome. Curr Genet..

[47] Zhao Z, Liu H, Wang C, Xu JR (2013). Comparative analysis of fungal genomes reveals different plant cell wall degrading capacity in fungi. BMC Genomics.

[48] Winnenburg R, Urban M, Beacham A, Baldwin TK, Holland S, Lindeberg M (2008). PHI-base update: additions to the pathogen host interaction database. Nucleic Acids Res.

[49] Li D, Chung KR, Smith DA, Schardl CL (1995). The *Fusarium solani* gene encoding kievitone hydratase a secreted enzyme that catalyzes detoxification of a bean phytoalexin. Mol Plant-Microbe Interact.

[50] Silva JC, Townsend CA (1997). Heterologous expression isolation and characterization of versicolorin B synthase from Aspergillus parasiticus a key enzyme in the aflatoxin B1 biosynthetic pathway. J Biol Chem.

[51] Toruño TY, Stergiopoulos I, Coaker G (2016). Plant-pathogen effectors: cellular probes interfering with plant defenses in spatial and temporal manners. Annu Rev Phytopathol.

[52] Caceres I, Al Khoury A, El Khoury R, Lorber S, Oswald IP, El Khoury A, Atoui A, Puel O, Bailly JD (2020). Aflatoxin biosynthesis and genetic regulation: A Review. Toxins.

[53] Westrick NM, Smith DL, Kabbage M (2021). Disarming the host: detoxification of plant defense compounds during fungal necrotrophy. Front Plant Sci.

[54] Hwang CS, Kolattukudy PE (1995). Isolation and characterization of genes expressed uniquely during appressorium formation by *Colletotrichum gloeosporioides* conidia induced by the host surface wax. Mol Gen Genet.

[55] Morrissey JP, Osbourn AE (1999). Fungal resistance to plant antibiotics as a mechanism of pathogenesis. Microbiol Mol Biol Rev.

[56] Segal LM, Wilson RA (2018). Reactive oxygen species metabolism and plant-fungal interactions. Fungal Genet Biol.

[57] Abdul W, Aliyu SR, Lin L, Sekete M, Chen X, Otieno FJ (2018). Family-four aldehyde dehydrogenases play an indispensable role in the pathogenesis of *Magnaporthe oryzae*. Front Plant Sci.

[58] Maloney A, VanEtten H (1994). A gene from the fungal plant pathogen *Nectria haematococca* that encodes the phytoalexin-detoxifying enzyme pisatin demethylase defines a new cytochrome P450 family. Mol Gen Genet.

[59] Tokai T, Koshino H, Takahashi-Ando N, Sato M, Fujimura M, Kimura M (2007). *Fusarium* Tri4 encodes a key multifunctional cytochrome P450 monooxygenase for four consecutive oxygenation steps in trichothecene biosynthesis. Biochem Biophys Res Commun.

[60] Sahu BB, Baumbach JL, Singh P, Srivastava SK, Yi X, Bhattacharyya MK (2017). Investigation of the *Fusarium virguliforme* transcriptomes induced during infection of soybean roots suggests that enzymes with hydrolytic activities could play a major role in root necrosis. PLoS One.

[61] Coleman JJ, Wasmann CC, Usami T, White GJ, Temporini ED, McCluskey K (2011). Characterization of the gene encoding pisatin demethylase (FoPDA1) in *Fusarium oxysporum*. Mol Plant-Microbe Interact.

[62] Cheah E, Ashley GW, Gary J, Oilis D (1993). Catalysis by dienelactone hydrolase: a variation on the protease mechanism. Proteins.

[63] Bowyer PBR, Clarke P, Lunness MJ, Osbourn AE (1995). Host range of a plant pathogenic fungus determined by a saponin detoxifying enzyme. Science.

[64] Zhang Y, Gao Y, Liang Y, Dong Y, Yang X, Yuan J, Qiu D (2017). The *Verticillium dahliae* SnodProt1-like protein VdCP1 contributes to virulence and triggers the plant immune system. Front Plant Sci.

[65] Modolo LV, Cunha FQ, Braga MR, Salgado I (2002). Nitric oxide synthase-mediated phytoalexin accumulation in soybean cotyledons in response to the *Diaporthe phaseolorum* f sp *meridionalis* elicitor. Plant Physiol.

[66] Lalitha B, Snow JP, Berggren GT (1989). Phytotoxin production by *Diaporthe phaseolorum* var *caulivora* the causal organism of stem canker of soybean. Phytopathology.

[67] Pietro AD, Huertas-González MD, Gutierrez-Corona JF, Martínez-Cadena G, Méglecz E, Roncero MIG (2001). Molecular characterization of a subtilase from the vascular wilt fungus *Fusarium oxysporum*. Mol Plant-Microbe Interact.

[68] Coleman JJ, Mylonakis E (2009). Efflux in fungi: La pièce de résistance. PLoS Pathog.

[69] Kolmogorov M, Yuan J, Lin Y, Pevzner PA (2019). Assembly of long error-prone reads using repeat graphs. Nat Biotechnol.

[70] Wick RR, Schultz MB, Zobel J, Holt KE (2015). Bandage: interactive visualization of de novo genome assemblies. Bioinformatics.

[71] Gurevich A, Saveliev V, Vyahhi N, Tesler G (2013). QUAST: quality assessment tool for genome assemblies. Bioinformatics.

[72] Min B, Grigoriev IV, Choi IG (2017). FunGAP: fungal genome annotation pipeline using evidence-based gene model evaluation. Bioinformatics.

[73] Stanke M, Keller O, Gunduz I, Hayes A, Waack S, Morgenstern B (2006). AUGUSTUS: Ab initio prediction of alternative transcripts. Nucleic Acids Res.

[74] Hoff KJ, Lange S, Lomsadze A, Borodovsky M, Stanke M (2016). BRAKER1: unsupervised RNA-Seq-based genome annotation with GeneMark-ET and AUGUSTUS. Bioinformatics.

[75] Cantarel BL, Korf I, Robb SMC, Parra G, Ross E, Moore B (2008). MAKER: an easy-to-use annotation pipeline designed for emerging model organism genomes. Genome Res.

[76] Bolger AM, Lohse M, Usadel B (2014). Trimmomatic: a flexible trimmer for Illumina sequence data. Bioinformatics.

[77] Simão FA, Waterhouse RM, Ioannidis P, Kriventseva EV, Zdobnov EM (2015). BUSCO: assessing genome assembly and annotation completetness with single-copy orthologs. Bioinformatics.

[78] Altschul SF, Gish W, Miller W, Myers EW, Lipman DJ (1990). Basic local alignment search tool. J Mol Biol.

[79] Zdobnov EM, Apweiler R (2001). InterProScan--an integration platform for the signature-recognition methods in InterPro. Bioinformatics.

[80] Güldener U, Mannhaupt G, Münsterkötter M, Haase D, Oesterheld M, Stümpflen V (2006). FGDB: a comprehensive fungal genome resource on the plant pathogen *Fusarium graminearum*. Nucleic Acids Res.

[81] Edgar RC (2004). MUSCLE: multiple sequence alignment with high accuracy and high throughput. Nucleic Acids Res.

[82] Nguyen LT, Schmidt HA, von Haeseler A, Minh BQ (2015). IQ-TREE: a fast and effective stochastic algorithm for estimating maximum-likelihood phylogenies. Mol Biol Evol.

[83] Kalyaanamoorthy S, Minh BQ, Wong TKF, von Haeseler A, Jermiin LS (2017). ModelFinder: fast model selection for accurate phylogenetic estimates. Nat Methods.

[84] Emms DM, Kelly S (2015). OrthoFinder: solving fundamental biases in whole genome comparisons dramatically improves orthogroup inference accuracy. Genome Biol.

[85] Petersen TN, Brunak S, Von Heijne G, Nielsen H (2011). SignalP 40: discriminating signal peptides from transmembrane regions. Nat Methods.

[86] Horton P, Park KJ, Obayashi T, Fujita N, Harada H (2007). WoLF PSORT: protein localization predictor. Nucleic Acids Res.

[87] Kall L, Krogh A, Sonnhammer ELL (2007). Advantages of combined transmembrane topology and signal peptide prediction - the Phobius web server. Nucleic Acids Res.

[88] do Amaral AM, Antoniw J, Rudd JJ, Hammond-Kosack KE. (2012). Defining the predicted protein secretome of the fungal wheat leaf pathogen *Mycosphaerella graminicola*. PLoS One.

[89] Kroghs A, Larsson B, von Heijne G, Sonnhammer ELL (2001). Predicting transmembrane protein topology with a hidden Markov model: application to complete genomes. J Mol Biol.

[90] de Castro E, Sigrist CJA, Gattiker A, Bulliard V, Langendijk-Genevaux PS, Gasteiger E (2006). ScanProsite: detection of PROSITE signature matches and ProRule associated functional and structural residues in proteins. Nucleic Acids Res.

[91] Sperschneider J, Gardiner DM, Dodds PN, Tini F, Covarelli L, Singh KB (2016). EffectorP: predicting fungal effector proteins from Secretomes using machine learning. New Phytol.

[92] Sperschneider J, Dodds PN, Gardiner DM, Singh KB, Taylor JM (2018). Improved prediction of fungal effector proteins from secretomes with EffectorP 20. Mol Plant Pathol.

[93] Sperschneider J, Dodds PN, Singh KB, Taylor JM (2017). ApoplastP: prediction of effectors and plant proteins in the apoplast using machine learning. New Phytol.

[94] Yin Y, Mao X, Yang J, Chen X, Mao F, Xu Y (2012). dbCAN: a web resource for automated carbohydrate-active enzyme annotation. Nucleic Acids Res.

[95] Kim D, Langmead B, Salzberg SL (2015). HISAT: a fast spliced aligner with low memory requirements. Nat Methods.

[96] Li H, Handsaker B, Wysoker A, Fennell T, Ruan J, Homer N (2009). 1000 genome project data processing subgroup the sequence alignment/map format and SAMtools. Bioinformatics.

[97] Liao Y, Smyth GK, Shi W (2013). FeatureCounts: an efficient general purpose program for assigning sequence reads to genomic features. Bioinformatics.

[98] Martini F, Binder H (2019). pcaExplorer: an R/bioconductor package for interacting with RNA-seq principal components. BMC Bioinform.

[99] Robinson MD, McCarthy DJ, Smyth GK (2009). edgeR: a bioconductor package for differential expression analysis of digital gene expression data. Bioinformatics.

[100] Oliveros JC. Venny: an interactive tool for comparing lists with Venn's diagrams. 2007-2015.https://bioinfogp.cnb.csic.es/tools/venny/index2.0.2.html.

