# Supplementary figures and images for: Comparative genomics of plant pathogenic Diaporthe species and transcriptomics of Diaporthe caulivora during host infection reveal insights into pathogenic strategies of the genus
Source: BMC Genomics. 2022 Mar 3;23:175. doi: 10.1186/s12864-022-08413-y (PMC8896106; doi:10.1186/s12864-022-08413-y)

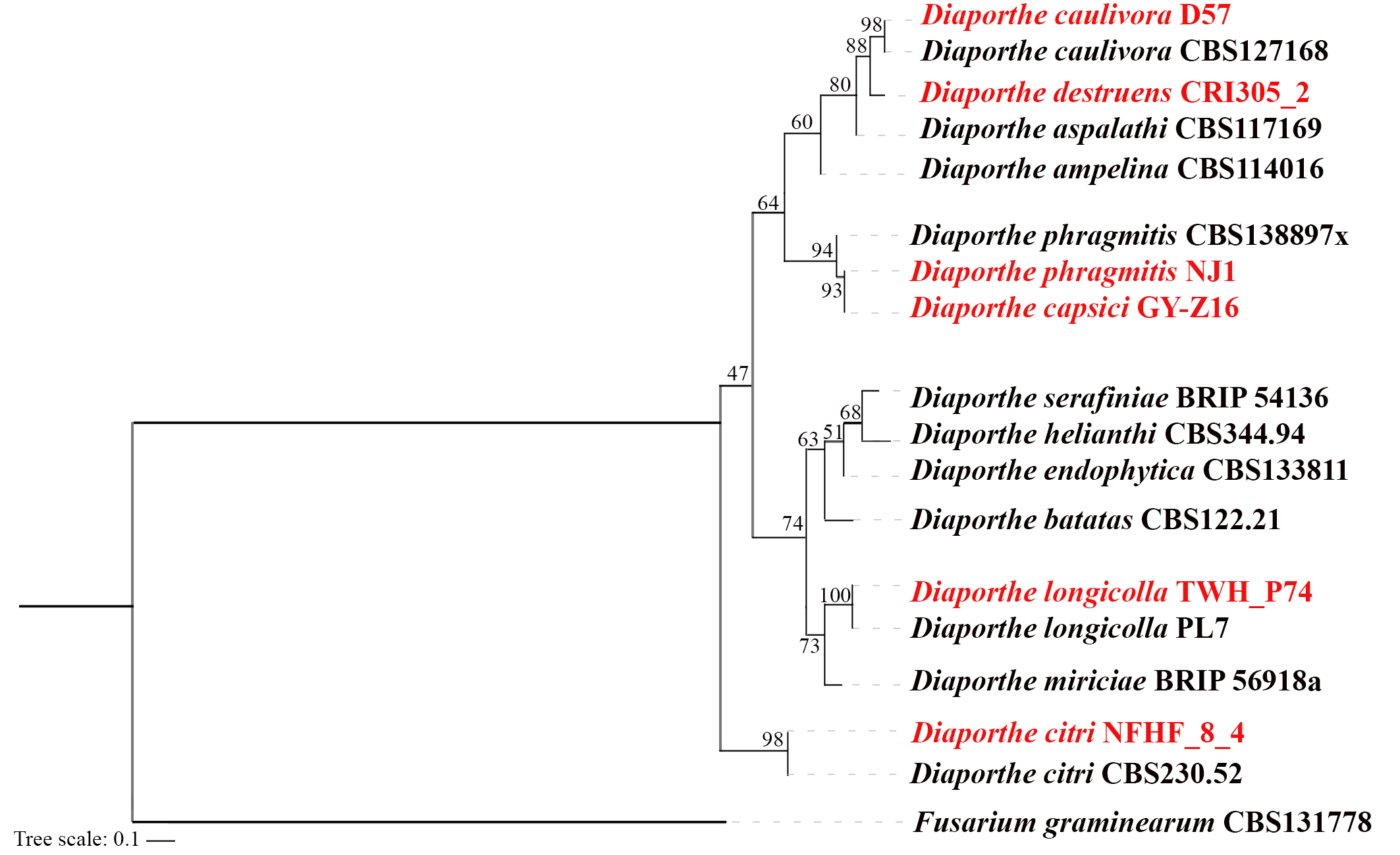

Supplement: Supplementary file 3 — Additional file 3. Phylogenetic tree generated from the analysis of internal transcribed spacer (ITS) and translation elongation factor 1-alpha gene (TEF1α) regions of the six Diaporthe species used in this study. The number at the branch nodes indicates bootstrap values (%) built on 1000 replications. The Diaporthe species used in this study are indicated in red and the ex-types strains in black. Fusarium graminearum was used as outgroup. ITS and TEF1α sequences of D. destruens were only obtained from the genome of strain CRI305_2 since sequences of other D. destruens strains were not available at NCBI. [file 12864_2022_8413_MOESM3_ESM.tif]

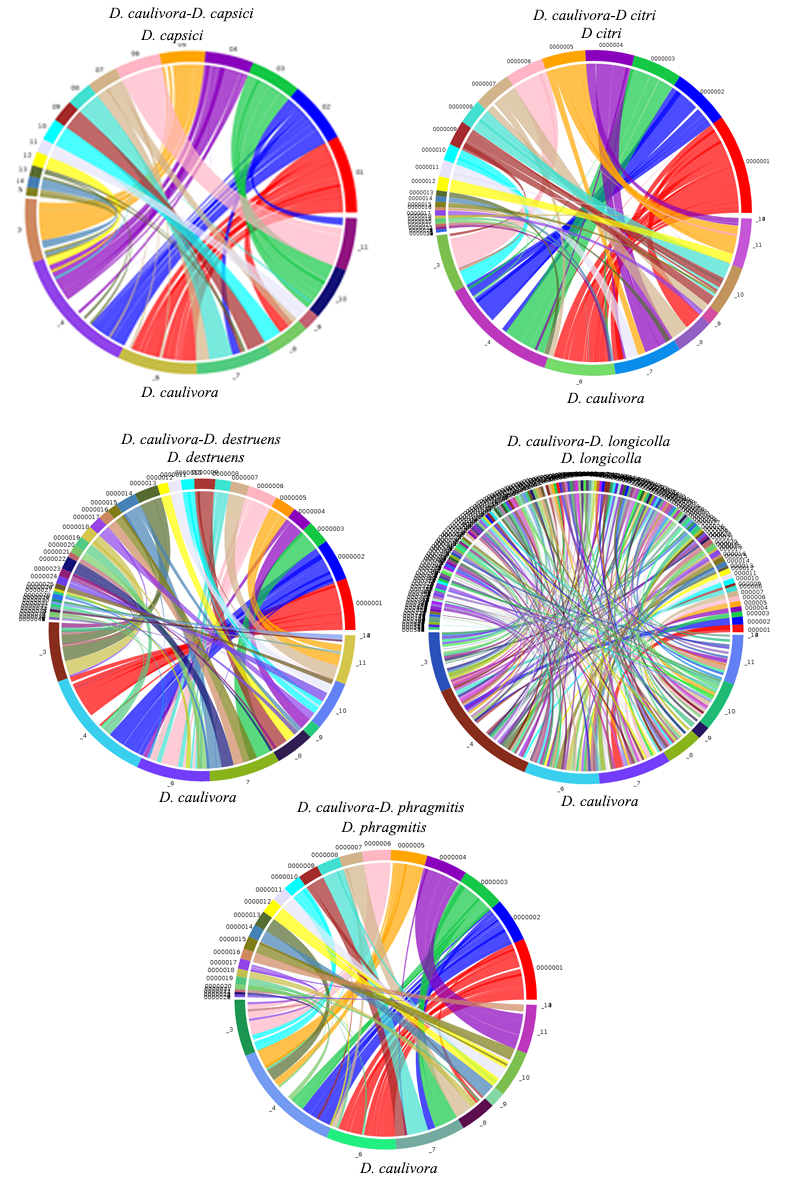

Supplement: Supplementary file 4 — Additional file 4. Synteny analysis between D. caulivora and other Diaporthe species. The synteny circle plots show the large synthenic blocks between D. caulivora and the other five Diaporthe species obtained using SyMAP. [file 12864_2022_8413_MOESM4_ESM.tif]
